# Clinical and Ultrasound Remission in Rheumatoid Arthritis Patients Treated with JAK Inhibitors: A Real-World Study

**DOI:** 10.3390/jcm15010278

**Published:** 2025-12-30

**Authors:** Carmen Lasa-Teja, Juan José Fernández-Cabero, Lara Sánchez-Bilbao, Javier Loricera, Iñigo González-Mazón, Carmen Álvarez-Reguera, Alba Herrero-Morant, Alfonso Corrales-Martínez, Virginia Portilla-González, Jose Luis Martín-Varillas, Laura Pérez-Garrido, Montserrat Santos-Gómez, Marcos López-Hoyos, Ricardo Blanco

**Affiliations:** 1 Department of Rheumatology, Hospital Universitario Marqués de Valdecilla, Immunopathology Group, Instituto de Investigación Valdecilla (IDIVAL), 39008 Santander, Spain; carmenlasa19@gmail.com (C.L.-T.); lasanbil@gmail.com (L.S.-B.); jmedtor@hotmail.com (J.L.); iglezmazon@gmail.com (I.G.-M.); alvarezreguera@gmail.com (C.Á.-R.); alhermor@gmail.com (A.H.-M.); afcorralesm@hotmail.com (A.C.-M.); virgiportilla@hotmail.com (V.P.-G.); 2 Division of Immunology, Hospital Universitario Marqués de Valdecilla, Instituto de Investigación Valdecilla (IDIVAL), 39008 Santander, Spain; juanfdezcabero@usal.es (J.J.F.-C.); marcos.lopez@scsalud.es (M.L.-H.); 3 Division of Rheumatology, Hospital Comarcal de Laredo, 39770 Laredo, Spain; jlmvarillas@gmail.com (J.L.M.-V.); pgarridolaura@gmail.com (L.P.-G.); 4 Division of Rheumatology, Hospital de Sierrallana, 39300 Torrelavega, Spain; montserrat.gomez@scsalud.es

**Keywords:** rheumatoid arthritis, clinical remission, DAS28-CRP, ultrasonographic remission, Janus kinase inhibitors, JAKI, disease activity, musculoskeletal ultrasound, Targeted Therapy, Treat-To-Target

## Abstract

**Background:** Janus kinase inhibitors (JAKi) are approved for the treatment of rheumatoid arthritis (RA), aiming to achieve clinical remission. Composite scores such as Disease Activity Score in 28 joints with C-reactive protein (DAS28-CRP) are influenced by subjective factors, and JAKi may impact these dimensions beyond inflammation. Ultrasound provides a sensitive and objective assessment of synovial activity. **Objective:** To evaluate clinical and ultrasound-defined remission in RA patients treated with JAKi under routine care. **Methods:** This cross-sectional study included all consecutive patients treated with baricitinib, filgotinib, tofacitinib, or upadacitinib between 1 November 2022 and 30 April 2023. Clinical remission was defined as DAS28-CRP and ultrasound remission as absence of power Doppler (PD) signal across a standardized 32-joint evaluation. **Results:** We include 78 patients with established RA; 87.2% were female, with mean age of 59.5 ± 10.8 years and disease duration of 10.6 ± 8.0 years. Most were seropositive for RF and/or ACPA (74.4%), and comorbidities were highly prevalent (93.6%). Clinical remission was observed in 42.3% and ultrasound remission in 56.4%, with no statistically significant differences between JAKi groups. Among 50 patients meeting remission by either definition, 30 (60%) fulfilled both criteria, 11 (22%) had ultrasound remission only, and 9 (18%) met clinical remission without sonographic confirmation. Discordant cases were often associated with osteoarthritis, fibromyalgia, mood disorders, and elevated inflammatory markers. **Conclusions:** JAKi were effective in achieving remission in many RA patients. Ultrasound revealed residual synovitis despite clinial remission and, conversely, silent remission in cases not meeting DAS28-CRP criterion, reinforcing its value for accurate monitoring and personalized therapeutic decisions. No meaningful clinical or ultrasonographic differences were observed between the various JAK inhibitors, indicating comparable perfomance across agents in routine practice.

## 1. Introduction

Rheumatoid arthritis (RA) is a chronic autoimmune disease characterized by persistent synovial inflammation, leading to joint destruction and extra-articular manifestations [1]. Although therapeutic advances have improved outcomes, achieving and sustaining remission remains a major challenge in clinical practice.

Conventional synthetic disease-modifying antirheumatic drugs (csDMARDs), such as methotrexate, remain the first-line treatment for RA [2,3]. However, many patients experience an inadequate response or intolerance [4]. Biologic DMARDs (bDMARDs)—including TNF inhibitors, IL-6 receptor antagonists, B-cell depleting agents, and T-cell co-stimulation modulators—have broadened therapeutic options [5,6]. More recently, Janus kinase inhibitors (JAKi), such as tofacitinib (TOFA), baricitinib (BARI), upadacitinib (UPA), and filgotinib (FILGO), have emerged as oral targeted synthetic DMARDs (tsDMARDs). These agents inhibit the JAK-STAT signaling pathway, thereby modulating key inflammatory cascades [7,8]. Notably, JAKi significantly impact pain and other patient-reported outcomes (PROs), enhancing clinical management from the patient’s perspective.

Despite these advances, remission remains elusive for many patients. In the ROADMAP cohort, only 37% maintained drug-free remission, and flares were common during DMARD tapering [9]. Additionally, a growing subset of patients with difficult-to-treat RA remains symptomatic despite sequential use of bDMARDs and JAKi [10,11,12,13]. These findings highlight the limitations of current strategies and the need for more precise tools to assess disease activity.

Clinical remission is commonly determined using composite indices such as the Disease Activity Score in 28 joints with C-reactive protein (DAS28-CRP), Clinical Disease Activity Index (CDAI), Simplified Disease Activity Index (SDAI), and Routine Assessment of Patient Index Data 3 (RAPID3) [12,13,14]. Nevertheless, these clinical indices frequently incorporate subjective elements—such as PROs and physician interpretation during joint examination—which may lead to misclassification, mistakenly categorizing patients as in remission or still exhibiting inflammation. Such discrepancies can have direct therapeutic implications, potentially leading to inappropriate escalation or the continuation of treatment [15,16].

In contrast, musculoskeletal ultrasound—particularly power Doppler imaging—not only provides a more accurate assessment of joint inflammation, but also confirms its absence, enabling the detection (or exclusion) of subclinical synovitis even in patients who meet clinical remission criteria [16,17]. Persistent Doppler signal has been linked to structural progression and increased risk of flare, while its absence correlates with better long-term outcomes [16,18]. These findings support the notion that ultrasonographic remission is useful both for identifying residual inflammation and verifying true remission [19,20]. Integrating ultrasound into the treat-to-target (T2T) strategy endorsed by EULAR may enhance disease monitoring and guide therapeutic decisions more effectively [21,22].

This study aims to evaluate the effectiveness of JAKi in achieving both clinical and ultrasound remission in RA patients under routine care and to assess the role of ultrasound in optimizing disease management.

## 2. Patients and Methods

### 2.1. Patients and Study Design

We conducted an observational, open-label study that included 78 consecutive patients diagnosed with RA from three rheumatology units in northern Spain treated with JAKi. All patients met the 2010 classification criteria established by the American College of Rheumatology (ACR) and the European Alliance of Associations for Rheumatology (EULAR) [2]. All patients were enrolled between 1 November 2022 and 30 April 2023.

Exclusion criteria included (a) age < 18 years; (b) absence of a confirmed RA diagnosis according to the 2010 ACR/EULAR criteria; (c) concomitant inflammatory arthritides; (d) incomplete clinical, laboratory or ultrasound data that precluded evaluation of remission status.

Most patients had previously received low- or moderate-dose glucocorticoids (GCs), along with csDMARDs and/or bDMARDs. All patients who had received at least one dose of JAKi were included, regardless of treatment outcome. JAKi were initiated either due to intolerance to previous therapies or refractoriness to csDMARDS and/or bDMARDs, as documented in the clinical record, following standard international recommendations, especially for the EULAR 2019 update for the management of rheumatoid arthritis and the ACR 2021 guideline for the treatment of RA [15].

Given the observational nature of the study, the sample size (*n* = 78) reflects the total eligible population during the recruitment period. This sample provides adequate precision to describe overall remission rates and ultrasound outcomes in routine clinical practice.

### 2.2. Clinical Definitions and Laboratory Data

Clinical remission was defined as the absence of signs and symptoms of significant inflammation, assessed using DAS28-CRP, with remission defined as a score < 2.6 [22].

Functional disability was assessed using the Health Assessment Questionnaire (HAQ), a validated patient-reported outcome measure widely used in rheumatoid arthritis. Scores range from 0 to 3, with higher values indicating greater disability. HAQ values were collected when available from routine clinical records.

Clinical joint counts were conducted by an independent assessor who was not involved in treatment decisions.

Laboratory parameters were collected at baseline and during follow-up, reflecting the study’s original design to allow early therapeutic adjustments. As no such modifications were ultimately required, follow-up subsequently continued with standard 3-month intervals. No clinically relevant fluctuations in laboratory parameters were observed during the monitoring period. These included neutrophils, lymphocytes, platelets, hemoglobin, transaminases, and serum creatinine. Inflammatory markers, including Erythrocyte sedimentation rate (ESR) and CRP, were measured at each visit. ESR above 20 mm/hour in men and above 25 mm/hour in women and CRP greater than 0.5 mg/dL were considered abnormal [17].

Rheumatoid factor (RF) and anti-cyclic citrullinated peptide antibodies (ACPAs) were assessed at baseline to characterize the serologic profile and explore associations with disease activity and treatment response. Disease activity was assessed by trained investigators using the core set of outcome measures developed by the OMERACT initiative and endorsed by EULAR [16].

### 2.3. Ultrasound Assessment

Musculoskeletal ultrasound was performed by a single experienced rheumatologist in ultrasound imaging with certified competence in musculoskeletal ultrasound, using a high-frequency linear transducer (6–18 MHz). Each examination lasted approximately 30 min and included a 32-joint assessment: extensor carpi ulnaris tendons, metacarpophalangeal and proximal interphalangeal joints, posterior tibial tendons, and 2nd–5th metatarsophalangeal joints.

Each joint was scored for power Doppler (PD) signal on a semi-quantitative scale from 0 to 3, reflecting vascularity within the synovium. The number of PD-positive joints and the total PD score (range: 0–96) were recorded. Synovial hypertrophy was scored in greyscale (GS) on semi-quantitative scale from 0 to 4. Ultrasound remission was defined as the absence of power Doppler signal (PD score = 0) in all evaluated joints, based on the EULAR–OMERACT combined scoring system for synovitis in RA [18].

Erosions attributable to osteoarthritis were excluded by requiring the absence of osteophytes, central erosions, joint-space narrowing patterns typical of osteoarthritis, and subchondral sclerosis. Erosive findings were only attributed to RA when they met OMERACT definitions for inflammatory bone erosion.

### 2.4. Data Collection and Ethics

Clinical, laboratory, and imaging data were extracted from electronic medical records by the investigators and entered into a standardized database. Information collected included treatment regimens, GC doses before and during JAKi therapy, and disease activity measures. The study was approved by the local Ethics Committee for Clinical Research of Cantabria, Spain (approval number: 2022.184).

### 2.5. Statistical Analysis

Continuous variables are expressed as mean ± standard deviation (SD) or median and interquartile range [IQR], as appropriate. Comparisons between groups were performed using Student’s *t*-test or the Kruskal–Wallis test for continuous variables and the chi-squared test for categorical variables. A *p*-value < 0.05 was considered statistically significant.

## 3. Results

### 3.1. Demographic and Baseline Characteristics

A total of 78 RA patients treated with JAKi were included: BARI (*n* = 34; 43.6%), FILGO (*n* = 19; 24.4%), TOFA (*n* = 14; 17.9%), and UPA (*n* = 11; 14.1%). The mean age was 59.5 ± 10.8 years, with BARI patients being significantly older (mean 63.4 years; *p* = 0.040). Most were women (*n* = 68, 87.2%), with a slightly lower female proportion in the BARI group (*n* = 26, 72.2%; *p* = 0.063).

RA duration averaged 10.6 ± 8.0 years, with no significant difference among groups (*p* = 0.277). Time from JAKi initiation varied significantly (*p* < 0.001), with TOFA patients having the longest treatment exposure (mean 46.7 ± 29.4 months), while FILGO users had only 5.4 ± 1.8 months.

Seropositivity rates were high: RF positive in 51 (71.8%) patients, and ACPA positive in 51 (65.4%) patients, with slightly higher ACPA rates among FILGO (*n* = 16, 84.2%) and UPA (*n* = 9, 81.8%) users, but without reaching statistical significance (*p* = 0.052). The combined RF/ACPA positivity was present in 58 (74.4%) patients. Extraarticular manifestations were uncommon (*n* = 8, 10.3%) and limited mainly to BARI and TOFA groups.

Erosive disease, identified by X-rays and also by US, was present in 27 (34.6%) of patients, without statistical differences between groups (*p* = 0.795). By treatment subgroup, erosions were identified in 10 patients receiving BARI (29.7%), 7 receiving FILGO (36.8%), 5 receiving TOFA (35.7%), and 5 receiving UPA (45.5%). Comorbidities were highly prevalent (*n* = 73, 93.6%), particularly in TOFA and UPA patients (100%).

Extra-articular manifestations included rheumatoid nodules (*n* = 1), secondary Sjögren’s syndrome (*n* = 4), Peripheral Ulcerative Keratitis (*n* = 1), and vasculitis (*n* = 1). These were observed mainly in the BARI and TOFA groups.

Comorbidities were highly prevalent (93.6%), including hypertension (46.2%), dyslipidemia (56.4%), type 2 diabetes (12.8%), endocrine disorders (9%), pulmonary disease (6.4%), and anxiety or depressive disorders (4%).

Baseline characteristics by JAKi group are presented in Table 1.

CRP levels were low across all groups, with a median of 0.4 [IQR: 0.4–0.7] mg/dL, and no significant differences observed (*p* = 0.490). Median ESR was 24.0 [IQR: 13–40.3] mm/h, with no significant intergroup differences (*p* = 0.063).

### 3.2. Treatment Exposure

Prednisone was used by 37 patients (47.4%) in the total cohort. By treatment group, 17 (50%) in BARI, 10 (52.6%) in FILGO, 7 (50%) in TOFA, and 3 (27.3%) in UPA were receiving glucocorticoids at the time of assessment. The median prednisone dose was 2.5 [IQR: 0–7.5] mg/day, with similar distribution across treatment groups (*p* = 0.591), with 20 patients (58.8%) remaining on low-dose glucocorticoids (≤7.5 mg/dar) and 14 (41.2%) on moderate doses (>7.5 mg/day). Before starting JAKi, 78 patients (100%) were receiving glucocorticoids.

Methotrexate was the most commonly used csDMARD (*n* = 19, 25.6%), with similar distribution across groups. Concomitant csDMARD use at JAKi initiation was observed in 37 patients (47.7%). Methotrexate monotherapy was the most frequent regimen (*n* = 20, 25.6%), followed by leflunomide in 7 (9%) and hydroxychloroquine in 6 (7.7%). Combination csDMARD therapy was less common (*n* = 4, 5.1%), including methotrexate plus hydroxychloroquine (*n* = 3) and hydroxychloroquine plus sulfasalazopyrine (*n* = 1).

Prior bDMARD exposure was common, with a median of 2 [IQR: 1–3] agents overall, and was evenly distributed across groups. The two most frequently used biologics were TNF inhibitors (62 patients, 79.5%) and IL-6 inhibitors (49 patients, 62.8%). TNF inhibitors previously used included adalimumab X = 36 (46.2%), etanercept X = 30 (38.5%), infliximab X = 13 (16.7%), X = 17 certolizumab pegol (21.8%), and X = 18 golimumab (23.1%). Other biologic therapies included Abatacept and Rituximab, which were used in smaller proportions. The distribution of previous bDMARD exposure was similar across JAK inhibitor groups, and no significant differences were observed in prior anti-TNF use (*p* = 0.684).

### 3.3. Clinical Remission

Remission according to DAS28-CRP was achieved in 42.3% of patients, with the highest rate in FILGO (61.1%), followed by TOFA (50.0%), BARI (40.0%), and UPA (27.3%), with no statistically significant differences between groups (*p* = 0.378). These findings are summarized in Table 2.

Regarding clinical relevance, FILGO and TOFA showed numerically higher remission rates, but the lack of statistical significance, the small subgroup sizes, and heterogeneity in treatment exposure prevent concluding that any specific JAK inhibitor is clinically more potent. Overall, no agent demonstrated a consistent clinical advantage in this cohort.

### 3.4. Ultrasound Remission

Ultrasound remission, defined as the complete absence of power Doppler signal, was achieved in 56.4% of patients. The highest rates were observed in TOFA (71.4%) and BARI (61.2%), followed by UPA (54.5%) and FILGO (42.1%). There were no statistically significant differences between groups (*p* = 0.135). These findings are illustrated in Table 2.

Ultrasound remission rates exceeded those of clinical indices such as DAS28-CRP. This discrepancy highlights the influence of patient-reported symptoms and the subjectivity inherent in clinical examination, particularly when functional or symptomatic measures such as the HAQ are considered.

**Figure 1:**
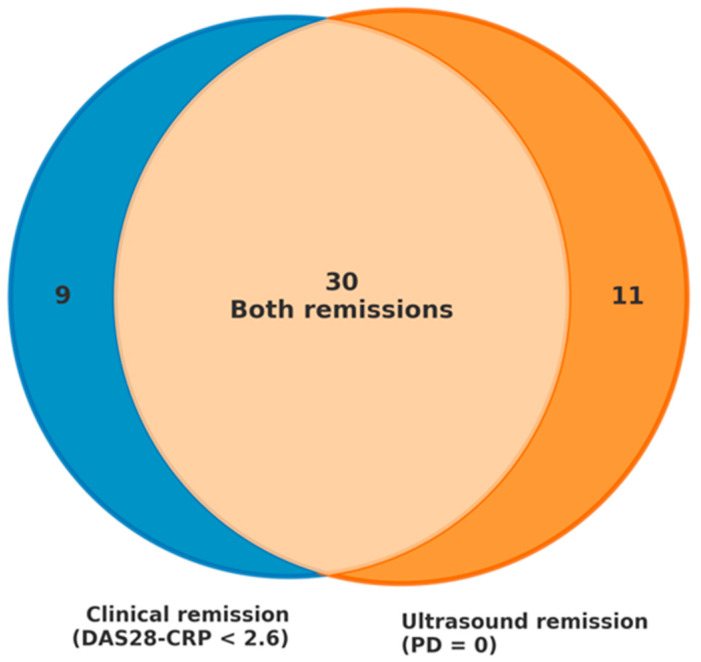
**Concordance between clinical and ultrasound remission.** Venn diagram illustrating the overlap between patients achieving clinical remission (by any index) and ultrasound remission (power Doppler score = 0). A subset of patients achieved ultrasound remission despite not fulfilling clinical remission criteria, highlighting the discordance between clinical and imaging assessments.

Table 2 summarizes remission outcomes across DAS28-CRP and ultrasound, while Figure 1 depicts their overlap and discordance.

Out of 78 patients, 50 achieved remission according to at least one criterion (DAS28-CRP < 2.6 or PD = 0 on ultrasound). Of those, 30 (60%) met both clinical and ultrasound remission criteria, 9 (18%) achieved clinical remission without ultrasound remission, and 11 (22%) fulfilled ultrasound remission criteria without meeting clinical remission thresholds. In this last subgroup of 11 patients, the following comorbidities and abnormalities were present: 5 (45.5%) had moderate-to-severe osteoarthritis, 2 (18.2%) had fibromyalgia, 4 (36.4%) presented with anxiety or depressive disorders, and 5 (45.5%) showed elevated acute-phase reactants. These comorbidities and biological markers may have contributed to the discrepancy between clinical indices and imaging findings, suggesting inactive synovitis despite residual symptoms or laboratory abnormalities.

### 3.5. Safety Profile

Safety outcomes were similar across all JAK inhibitors. The overall incidence of adverse events was low, with no significant differences between groups in infections, laboratory abnormalities, treatment discontinuations, cardiovascular events, or venous thromboembolism. No major cardiovascular or thromboembolic events were observed during follow-up. The frequency and pattern of adverse events did not indicate superior safety or higher toxicity for any JAK inhibitor.

## 4. Discussion

This real-world study provides valuable insights into the effectiveness of JAKi in achieving both clinical and ultrasonographic remission in patients with long-standing RA, many of whom had previously failed multiple lines of therapy. Our findings reinforce the therapeutic potential of JAKi in difficult-to-treat populations and highlight the added value of musculoskeletal ultrasound—not only in detecting residual synovial inflammation that may be missed by conventional clinical indices, but also in confirming the absence of active disease in patients with persistent symptoms unrelated to inflammation. This dual role is fundamental for reducing misclassification and guiding more accurate, individualized therapeutic decisions [8,9,10].

A central observation in our cohort was the discordance between clinical and imaging remission. While clinical remission rates were 42.3% according to DAS28-CRP, ultrasound remission—defined as the complete absence of power Doppler signal—was achieved in 56.4% of patients. Such divergence highlights that some patients not meeting clinical criteria might still be in true remission according to imaging. This finding has been consistently reported in the literature [11,12,13]. It underscores an important limitation of relying exclusively on clinical composite scores, as these may be influenced by subjective factors such as pain perception, fatigue, or comorbidities including fibromyalgia [14].

A key observation in the discordance between clinical and ultrasound-based remission was that, of the 50 patients who achieved remission by either method, 22% (11 patients) showed ultrasound remission despite not meeting clinical criteria, while 18% (9 patients) met DAS28-CRP remission thresholds but still had Doppler signal indicating active synovitis. Overall, nearly 40% of patients were classified differently depending on the assessment modality. This has relevant clinical implications: without ultrasound, some patients could be considered to have active disease and receive unnecessary treatment escalation, potentially increasing the risk of adverse effects and healthcare costs without clinical benefit. Ultrasound offers a more objective evaluation of disease activity, helping to distinguish between inflammatory activity and non-inflammatory symptoms [15,16], and its integration into routine assessment may reduce overtreatment and support more individualized, evidence-based therapeutic decisions [16].

Another noteworthy finding was the differential performance of JAKi across assessment tools. FILGO was associated with the highest rates of clinical remission, according to DAS28-CRP, while TOFA and BARI were more frequently associated with ultrasound remission. These differences may reflect distinct pharmacokinetic and pharmacodynamic properties among JAKi, including selectivity for JAK isoforms, tissue penetration, and cytokine modulation profiles [4,5,6,7,18]. For instance, TOFA and BARI inhibit both JAK1 and JAK3 or JAK2, respectively, which may result in broader immunomodulatory effects, potentially more evident at the microvascular level captured by Doppler imaging [19]. In contrast, FILGO is a selective JAK1 inhibitor, which may favor symptom control with a different impact on synovial vascularity [23].

The safety profile in our cohort was favorable, with no serious adverse events reported. This aligns with findings from pivotal trials and long-term extension studies [4,5,6,7], although retrospective designs may underestimate adverse events due to underreporting. Importantly, recent updates have highlighted the need for individualized risk assessment when initiating JAK inhibitors [19,20]. This is particularly relevant in patients with cardiovascular risk factors or history of malignancy.

Our findings support the integration of musculoskeletal ultrasound into routine RA management. Beyond its diagnostic utility, ultrasound provides a dynamic and objective assessment of synovial inflammation, enabling a more precise evaluation of treatment response and remission status [9,16]. In patients who meet clinical remission criteria but still exhibit Doppler activity, treatment intensification may be warranted to prevent structural progression. Conversely, in patients with no Doppler signal—indicating true imaging remission—tapering strategies could be considered with greater confidence [22]. Thus, ultrasound may serve as a valuable tool in guiding personalized, treat-to-target approaches.

This study has several limitations. The retrospective design and relatively small sample size, particularly when stratified by treatment group, limit the statistical power and generalizability of our findings. The absence of longitudinal ultrasound data precludes any assessment of the durability of imaging remission and its correlation with long-term outcomes. Additionally, the lack of standardized ultrasound scoring systems across centers may introduce variability in interpretation. Future prospective studies with larger cohorts, standardized imaging protocols, and long-term follow-up are needed to validate these observations and explore the prognostic value of combined clinical and imaging remission [24,25].

The safety profile in our cohort was favorable, with no serious adverse events reported. This is consistent with the results of pivotal trials and long-term extension studies [4,5,6,7], although the retrospective nature of our design may underestimate adverse events due to underreporting. Importantly, recent regulatory and scientific updates emphasize the need for individualized risk assessment when initiating JAK inhibitors [20,21]. This is particularly relevant in patients with cardiovascular risk factors or a history of malignancy, underscoring the importance of careful patient selection and ongoing safety monitoring.

In conclusion, JAKi are effective in achieving both clinical and ultrasonographic remission in patients with established RA. The use of musculoskeletal ultrasound enhances disease assessment by identifying subclinical inflammation and confirming true remission, helping to avoid unnecessary treatment escalation. Integrating imaging into routine care may support more personalized and accurate management strategies.

## Figures and Tables

**Table 1:** Characteristics of RA patients by JAKi at the moment of study. Data are presented as mean ± SD, median (IQR), or number (percentage), as appropriate. *p*-values refer to comparisons across the four JAK inhibitor groups (baricitinib, filgotinib, tofacitinib, and upadacitinib). Statistical comparisons were performed using the Kruskal–Wallis test for continuous variables and the chi-squared test for categorical variables.

Variable	Total (N = 78)	BARI (*n* = 34)	FILGO (*n* = 19)	TOFA (*n* = 14)	UPA (*n* = 11)	*p*-Value
Demographics						
Age, years, mean ± SD	59.5 ± 10.8	63.4 ± 9.7	61.0 ± 11.4	55.5 ± 8.6	61.0 ± 12.5	**0.040**
Women, *n* (%)	68 (87.2)	26 (72.2)	18 (94.7)	14 (100)	11 (100)	0.063
Disease Characteristics						
RA duration, months, mean ± SD	126.6 ± 103.9	131.7 ± 123.6	169.5 ± 84.3	119.1 ± 62.6	87.2 ± 104.9	0.277
Months from JAKi initiation, mean ± SD	17.5 ± 20.1	14.0 ± 11.0	5.4 ± 1.8	46.7 ± 29.4	9.7 ± 5.0	**<0.001**
RF positive, *n* (%)	56 (71.8)	28 (82.4)	14 (73.7)	9 (64.3)	6 (54.5)	0.266
RF and/or ACPA positive, *n* (%)	58 (74.4)	23 (67.6)	17 (89.5)	10 (71.4)	8 (72.7)	0.368
ACPA positive, *n* (%)	51 (65.4)	20 (58.8)	16 (84.2)	6 (42.9)	9 (81.8)	0.052
Extraarticular manifestations, *n* (%)	8 (10.3)	6 (17.6)	0	2 (14.3)	0	0.128
Erosive disease, *n* (%)	27 (34.6)	10 (29.4)	7 (36.8)	5 (35.7)	5 (45.5)	0.795
Comorbidities, *n* (%)	73 (93.6)	33 (97.1)	18 (94.7)	14 (100)	11 (100)	0.173
Current Treatment						
Prednisone dose, mg/day, median [IQR]	2.5 (0–7.5)	2.5 (0–5)	2.5 (0–10)	2.5 (0–15)	7.5 (0–10)	0.591
NSAID use, *n* (%)	35 (44.9)	11 (32.3)	6 (31.6)	9 (64.3)	9 (81.8)	**0.008**
MTX use, *n* (%)	20 (25.6)	10 (29.4)	4 (21.1)	4 (28.6)	2 (18.2)	0.095
Other csDMARDs (leflunomide, sulfasalazine, and hydroxychloroquine), *n* (%)	19 (24.4)	7 (20.6)	6 (55.9)	4 (28.6)	2 (18.2)	0.094
Previous Treatment						
Previous anti-TNF, *n* (%)	62 (79.5)	27 (79.4)	14 (73.7)	13 (92.9)	8 (72.7)	0.779
Previous anti-IL-6, *n* (%)	49 (62.8)	21 (61.8)	14 (73.7)	8 (57.1)	6 (54.5)	0.684
Previous csDMARDs, median [IQR]	2 (2–3)	2 (2–3)	3 (1–4)	1.5 (1–4)	2 (1–4)	0.234
Previous bDMARDs, median [IQR]	2 (1–3)	2 (1–3)	2 (0–5)	2 (1–5)	3 (0–6)	0.749
Inflammatory Markers						
CRP (mg/dL, median [IQR])	0.4 (0.4–0.7)	0.4 (0.4–0.7)	0.4 [0.4–2.9]	0.5 (0.1–0.5)	0.4 (0.1–1.4)	0.490
ESR (mm/h, median [IQR])	24.0 (13.0–40.3)	21.5 (14–39.8)	17.0 (8–27)	24 (18–45)	31.5 (24–64)	0.063

Abbreviations (alphabetical order): ACPA: anti-citrullinated protein antibodies; BARI: baricitinib; bDMARDs: biological disease-modifying antirheumatic drugs; CRP: C-reactive protein; csDMARDs: conventional synthetic disease-modifying antirheumatic drugs; ESR: erythrocyte sedimentation rate; FILGO: filgotinib; MTX: methotrexate; NSAID: non-steroidal anti-inflammatory drug; RA: rheumatoid arthritis; RF: rheumatoid factor; TOFA: tofacitinib; UPA: upadacitinib. Values in bold indicate statistical significance (*p* < 0.05).

**Table 2:** Clinical and ultrasound remission rates by treatment group. Statistical comparisons were performed using the Kruskal–Wallis test for continuous variables and the chi-squared test for categorical variables.

Disease Activity and Remission	Total (N = 78)	BARI (*n* = 34)	FILGO (*n* = 19)	TOFA (*n* = 14)	UPA (*n* = 11)	*p*
DAS28-CRP remission, *n* (%)	33 (42.3)	12 (40.0)	11 (61.1)	7 (50.0)	3 (27.3)	0.378
DAS28-CRP low activity, *n* (%)	20 (25.6)	10 (33.3)	2 (11.1)	3 (21.4)	5 (45.5)	0.104
Ultrasound remission (PD = 0), *n* (%)	44 (56.4)	21 (61.2)	8 (42.1)	10 (71.4)	6 (54.5)	0.135

Abbreviations (alphabetical order): PD: power Doppler; BARI = baricitinib; DAS28-CRP = Disease Activity Score in 28 joints using C-reactive protein; FILGO = filgotinib; TOFA = tofacitinib; UPA = upadacitinib.

## Data Availability

The data presented in this study are available on request from the corresponding author. The data are not publicly available due to privacy and ethical restrictions.

## References

[1] Smolen J.S., Landewé R.B.M., Bergstra S.A., Kerschbaumer A., Sepriano A., Aletaha D., Caporali R., Edwards C.J., Hyrich K.L., Pope J.E. (2023). EULAR recommendations for the management of rheumatoid arthritis with synthetic and biological disease-modifying antirheumatic drugs: 2022 update. Ann. Rheum. Dis..

[2] Aletaha D., Neogi T., Silman A.J., Funovits J., Felson D.T., Bingham C.O., Birnbaum N.S., Burmester G.R., Bykerk V.P., Cohen M.D. (2010). 2010 Rheumatoid arthritis classification criteria: An American College of Rheumatology/European League Against Rheumatism collaborative initiative. Arthritis Rheum..

[3] Van der Heijde D., Klareskog L., Rodriguez-Valverde V., Codreanu C., Bolosiu H., Melo-Gomes J., Tornero-Molina J., Wajdula J., Pedersen R., Fatenejad S. (2006). Comparison of etanercept and methotrexate, alone and combined, in the treatment of rheumatoid arthritis: Two-year clinical and radiographic results from the TEMPO study, a double-blind, randomized trial. Arthritis Rheum..

[4] Taylor P.C., Keystone E.C., van der Heijde D., Weinblatt M.E., Del Carmen Morales L., Gonzaga J.R., Yakushin S., Ishii T., Emoto K., Beattie S. (2017). Baricitinib versus Placebo or Adalimumab in Rheumatoid Arthritis. N. Engl. J. Med..

[5] Genovese M.C., Fleischmann R., Combe B., Hall S., Rubbert-Roth A., Zhang Y., Zhou Y., Mohamed M.-E.F., Meerwein S., Pangan A.L. (2018). Safety and efficacy of upadacitinib in patients with active rheumatoid arthritis refractory to biologic disease-modifying anti-rheumatic drugs (SELECT-BEYOND): A double-blind, randomised controlled phase 3 trial. Lancet.

[6] Combe B., Kivitz A., Tanaka Y., van der Heijde D., Simon J.A., Baraf H.S.B., Kumar U., Matzkies F., Bartok B., Ye L. (2021). Filgotinib versus placebo or adalimumab in patients with rheumatoid arthritis and inadequate response to methotrexate: A phase III randomised clinical trial. Ann. Rheum. Dis..

[7] Van Vollenhoven R.F., Fleischmann R., Cohen S., Lee E.B., Meijide J.A.G., Wagner S., Forejtova S., Zwillich S.H., Gruben D., Koncz T. (2012). Tofacitinib or adalimumab versus placebo in rheumatoid arthritis. N. Engl. J. Med..

[8] Naredo E., Möller I., Cruz A., Carmona L., Garrido J. (2008). Power Doppler ultrasonographic monitoring of response to anti-tumor necrosis factor therapy in patients with rheumatoid arthritis. Arthritis Rheum..

[9] Rizzo C., Ceccarelli F., Gattamelata A., Vavala C., Valesini G., Iagnocco A. (2013). Ultrasound in rheumatoid arthritis. Med. Ultrason..

[10] Mandl P., Naredo E., Wakefield R.J., Conaghan P.G., D’Agostino M.A., OMERACT Ultrasound Task Force (2011). A systematic literature review analysis of ultrasound joint count and scoring systems to assess synovitis in rheumatoid arthritis according to the OMERACT filter. J. Rheumatol..

[11] Foltz V., Gandjbakhch F., Etchepare F., Rosenberg C., Tanguy M.L., Rozenberg S., Bourgeois P., Fautrel B. (2012). Power Doppler ultrasound, but not low-field magnetic resonance imaging, predicts relapse and radiographic disease progression in rheumatoid arthritis patients with low levels of disease activity. Arthritis Rheum..

[12] Brown A.K., Conaghan P.G., Karim Z., Quinn M.A., Ikeda K., Peterfy C.G., Hensor E., Wakefield R.J., O’Connor P.J., Emery P. (2008). An explanation for the apparent dissociation between clinical remission and continued structural deterioration in rheumatoid arthritis. Arthritis Rheum..

[13] Saleem B., Brown A.K., Keen H., Nizam S., Freeston J., Wakefield R., Karim Z., Quinn M., Hensor E., Conaghan P.G. (2011). Should imaging be a component of rheumatoid arthritis remission criteria? A comparison between traditional and modified composite remission scores and imaging assessments. Ann. Rheum. Dis..

[14] Rau R. (2006). Is remission in rheumatoid arthritis associated with radiographic healing?. Clin. Exp. Rheumatol..

[15] Terslev L., Ostergaard M. (2021). Rheumatoid Arthritis Relapse and Remission—Advancing Our Predictive Capability Using Modern Imaging. J. Inflamm. Res..

[16] D’Agostino M.A., Terslev L., Aegerter P., Backhaus M., Balint P., Bruyn G.A., Filippucci E., Grassi W., Iagnocco A., Jousse-Joulin S. (2017). Scoring ultrasound synovitis in rheumatoid arthritis: A EULAR-OMERACT ultrasound taskforce-Part 1: Definition and development of a standardised, consensus-based scoring system. RMD Open.

[17] Haavardsholm E.A., Aga A.B., Olsen I.C., Lillegraven S., Hammer H.B., Uhlig T., Fremstad H., Madland T.M., Lexberg Å.S., Haukeland H. (2016). Ultrasound in management of rheumatoid arthritis: ARCTIC randomised controlled strategy trial. BMJ.

[18] O’Shea J.J., Schwartz D.M., Villarino A.V., Gadina M., McInnes I.B., Laurence A. (2015). The JAK-STAT pathway: Impact on human disease and therapeutic intervention. Annu. Rev. Med..

[19] Schwartz D.M., Kanno Y., Villarino A., Ward M., Gadina M., O’Shea J.J. (2017). JAK inhibition as a therapeutic strategy for immune and inflammatory diseases. Nat. Rev. Drug Discov..

[20] Ytterberg S.R., Bhatt D.L., Mikuls T.R., Koch G.G., Fleischmann R., Rivas J.L., Germino R., Menon S., Sun Y., Wang C. (2022). Cardiovascular and Cancer Risk with Tofacitinib in Rheumatoid Arthritis. N. Engl. J. Med..

[21] (2023). Risk of serious adverse effects with Janus kinase inhibitors. Drug Ther. Bull..

[22] Takase-Minegishi K., Horita N., Kobayashi K., Yoshimi R., Kirino Y., Ohno S., Kaneko T., Nakajima H., Wakefield R.J., Emery P. (2018). Diagnostic test accuracy of ultrasound for synovitis in rheumatoid arthritis: Systematic review and meta-analysis. Rheumatology.

[23] Raimondo V., Caminiti M., Olivo D., Gigliotti P., L’andolina M., Muto P., Pellegrini R., Varcasia G., Bruno C., Massaro L. (2024). Real-Life Use of Filgotinib in Rheumatoid Arthritis: A Retrospective Cohort Study. J. Clin. Med..

[24] Colebatch A.N., Edwards C.J., Østergaard M., Van Der Heijde D., Bálint P.V., D’Agostino M.-A., Forslind K., Grassi W., Haavardsholm E.A., Haugeberg G. (2013). EULAR recommendations for the use of imaging of the joints in the clinical management of rheumatoid arthritis. Ann. Rheum. Dis..

[25] Wakefield R.J., D’Agostino M.A., Iagnocco A., Filippucci E., Backhaus M., Scheel A.K., Joshua F., Naredo E., A Schmidt W., Grassi W. (2007). The OMERACT Ultrasound Group: Status of current activities and research diections. J. Rheumatol..

